# MicroRNAs: a crossroad that connects obesity to immunity and aging

**DOI:** 10.1186/s12979-022-00320-w

**Published:** 2022-12-14

**Authors:** Ahmed Rakib, Sonia Kiran, Mousumi Mandal, Udai P. Singh

**Affiliations:** Department of Pharmaceutical Sciences, College of Pharmacy, The University of Tennessee Health Science Center, 881 Madison Avenue, Memphis, TN 38163 USA

**Keywords:** Obesity, miRs, Adipose tissue, Aging

## Abstract

Obesity is characterized by an elevated amount of fat and energy storage in the adipose tissue (AT) and is believed to be the root cause of many metabolic diseases (MDs). Obesity is associated with low-grade chronic inflammation in AT. Like obesity, chronic inflammation and MDs are prevalent in the elderly. The resident immune microenvironment is not only responsible for maintaining AT homeostasis but also plays a crucial role in stemming obesity and related MDs. Mounting evidence suggests that obesity promotes activation in resident T cells and macrophages. Additionally, inflammatory subsets of T cells and macrophages accumulated into the AT in combination with other immune cells maintain low-grade chronic inflammation. microRNAs (miRs) are small non-coding RNAs and a crucial contributing factor in maintaining immune response and obesity in AT. AT resident T cells, macrophages and adipocytes secrete various miRs and communicate with other cells to create a potential effect in metabolic organ crosstalk. AT resident macrophages and T cells-associated miRs have a prominent role in regulating obesity by targeting several signaling pathways. Further, miRs also emerged as important regulators of cellular senescence and aging. To this end, a clear link between miRs and longevity has been demonstrated that implicates their role in regulating lifespan and the aging process. Hence, AT and circulating miRs can be used as diagnostic and therapeutic tools for obesity and related disorders. In this review, we discuss how miRs function as biomarkers and impact obesity, chronic inflammation, and aging.

**Graphical Abstract:**

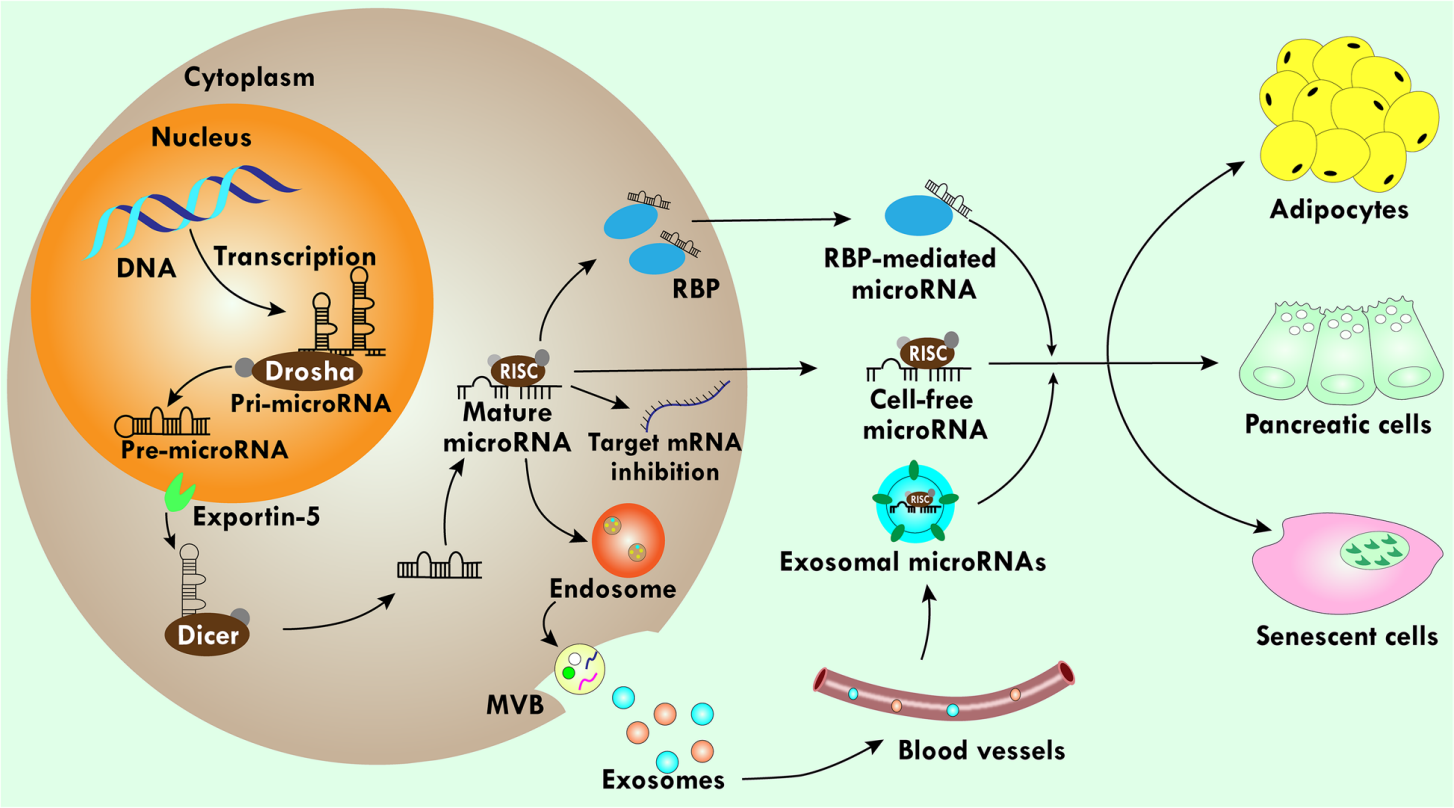

## Introduction

Obesity is a multifactorial disease condition characterized by low-grade chronic inflammation in the adipose tissue (AT) that adversely affects almost all physiological and biological functions of the body. The overall global number of obese and overweight persons is twice that of obese and overweight persons 50 years ago, and the World Health Organization (WHO) has estimated that almost three million people die each year due to obesity-associated complications [1]. According to the Centers for Disease Control and Prevention (CDC), the US obesity prevalence was shown at 41.9% from 2017 to 2020, and severe obesity doubled during this same period [2]. Emerging evidence over the last two decades has reported that obesity is associated with metabolic syndrome (MS), influences the immune system, and promotes several metabolic disorders including types 1 and 2 diabetes mellitus (T1 & T2DM), cardiovascular diseases (CVD), atherosclerosis, and musculoskeletal disorders [3–5]. Further, obesity mainly results from the impairment of the body’s energy balance system, which leads to the formation and accumulation of an excessive amount of fat in the AT [6]. This excessive amount of energy is transformed into a form of triglycerides and is stored in AT depots, while adipocytes proliferate and eventually expand, leading to an increased amount of body fat in the AT and ultimately resulting in weight gain [7].

Obesity is marked by increased production of proinflammatory cytokines and infiltration of immune cells including macrophages and T cells [8, 9]. The increased production of cytokines such as tumor necrosis factor (TNF)-α released by these immune cells further impairs metabolism and other aspects during obesity, resulting in metabolic organ dysfunction [8]. Although MS is not purely a metabolic disorder, it is a chronic systemic inflammatory syndrome characterized by an increased level of proinflammatory cytokines as well as the infiltration of macrophages into AT. Thereby, macrophages are the most crucial cell type dominant for immunometabolism, obesity-related tissue remodeling, and low-grade chronic inflammation [10]. It has been well established that macrophage frequency is quantitatively higher in obese animals compared to lean mice and AT macrophages represent specific cellular localization along with inflammatory potential [11]. To this end, obese AT macrophages are mostly dominated by the proinflammatory classical (M1) phenotype, whereas lean AT possesses an alternative (M2) phenotype [12]. In addition, mounting evidence suggests that T-cell infiltration followed by macrophage influx is strongly implicated in the initiation of AT inflammation associated with obesity [13, 14]. In obesity and T2DM, special subsets of T cells such as regulatory T cells (Tregs) are decreased in the AT [15]. On the other hand, effector T cells, comprising CD4 and CD8 T cells, T helper (Th)1 cell, and Th17 cells showed an upward trend during obesity and related MS [16]. Further, macrophages are at the frontier during the development of obesity and change their phenotypes according to the severity of obesity [17]. In both mice and humans, AT is infiltrated by macrophages during the progression of obesity [18]. During obesity, AT M1 macrophage numbers increase and correlate with AT inflammation and insulin resistance (IR) [19].

While looking at the molecular mechanism behind obesity-induced metabolic diseases (MDs), microRNAs (miRs) received immense attention in the last two decades and interestingly came out as a top mediator of obesity and MDs [20]. miRs are single-stranded RNA (ssRNAs) of 19–25 nucleotide length and generated mainly by Dicer belongs to an endogenous transcript, containing a local hairpin structure [21]. Functionally, miRs exert as guide molecules for post-transcriptional gene silencing by base pairing with specific messenger RNA (mRNA) [22]. After maturation, miRs are complexed with argonaute2 (AGO2), which worked as a slicer enzyme to cleave the target mRNA. Additionally, miRs are also packaged within extracellular vesicles (EVs) such as microvesicles or exosomes. Exosomes are membrane-bound vesicles that are released from almost all cell types and important for exchanging cellular information [23]. It has been shown that miRs proportion is higher in exosomes than in their parent cells and these cells exhibit a sorting mechanism to provide a sufficient guide for specific miRs to enter exosomes [24]. miRs are expressed by many peripheral tissues including AT, can regulate inflammation and MDs, and have been reported to differentially regulate the expression of target genes [25]. Moreover, miRs are associated with the proliferation and differentiation of adipocytes, which are a precursor for several proinflammatory cytokines and, notably, the expression of several signaling proteins associated with MS [26]. While there are currently few effective treatment strategies for obesity and related disorders, miRs have shown great therapeutical potential and can be utilized as both prognostic and diagnostic tools for obesity [27]. The differential expression of miRs in obesity and MDs is currently raising concern because miRs act differently during the progression of adiposity and disease severity [28]. Therefore, the investigation of miRs and their regulation patterns in several conventional and novel signaling pathways involved in the complex processes of obesity and MDs might pave the way for better understanding and designing future therapeutic approaches for obesity. For this reason, miRs have been recently emphasized as novel tools for therapeutic options for obesity and related diseases. Since miRs are associated with the expression and repression of target genes in cellular senescence, they also affect aging.

Taken together, this review is mainly focused on a crossroad that connects obesity to immunity through miRs regulation in AT through the infiltration of T cells and macrophage. This infiltration of immune cells induces low-grade chronic inflammation in the AT and sustains obesity, which drives the way toward aging. Additionally, we highlight the potential role of circulating miRs as potential biomarkers and therapeutic options for obesity and related MDs. In addition, we will also briefly discuss how these miRs alter aging through chronic inflammation in AT, all of which connects obesity conditions with several MDs.

### Contribution of immune cells to obesity

AT is considered a major crosstalk hub between immunity, metabolism, and nutrition and is regarded as a central mechanism that connects obesity with vascular and metabolic complications. AT is comprised of mature fat cells known as adipocytes, and a stromal vascular fraction, which consists of pre-adipocytes, fibroblasts, and immune cells [29]. Functionally, adipocytes synthesize and store triglycerides, and also hydrolyze and release triglycerides as a form of free fatty acids [30]. During obesity, adipocytes also function as endocrine cells and secrete adipokines, chemokines, and cytokines such as monocyte chemoattractant protein-1 (MCP-1) and TNF-α [31]. The MCP-1 production by adipocytes contributes to a proinflammatory state and these are also responsible for macrophage infiltration [32]. AT inflammation accompanies hypertrophic adipocytes along with infiltrating immune cell populations, resulting in an excess level of proinflammatory cytokines [33]. The AT resident immune cells include M1 macrophages, neutrophils, mast cells, CD4 and CD8 T cells, which all contribute to inflammation and IR. In contrast, certain immune cell attenuates inflammatory responses such as M2 macrophages, Tregs, and Th2 cells [34]. AT macrophage polarization of M1 and M2 phenotypes orchestrate the obese condition, through cytokines expression like IL-4 by M2, whereas M1 induces TNF-α, IL-6, and iNOS. Dendritic cells (DCs) are antigen-presenting cells that are expressed by CD11b, CD11c, and other co-stimulatory molecules [35]. It has been shown that the recruitment of DCs in adipose tissues required higher expression of the CCR7 receptor, and low CCR2 expression in HFD-induced obesity [36]. DCs also present in AT mediates Th17 response by IL-1β, IL-6, and IL-23 [36]. Hence, DCs are also considered an effective player in obesity and could be the origin of several obesity-related disorders.

Although most studies feature the inclusion of innate immune cells as a key player during obesity, recent studies also suggest a role of T-cell and B-cell-mediated humoral immunity in obesity. The obese adipocytes express abundant major histocompatibility complex (MHC) class II molecules and thus promoting CD4 T cell activation [37]. Further, obesity increases the frequency of both CD4 and CD8 T cells and the reduction of CD8 T cells significantly lowers the M1 macrophages [13]. Likewise, CD4 cells secreted cytokines mediate obesity and related metabolic complications [38]. Tregs also play a crucial role during obesity, and it has been shown that Tregs population frequency decreased during obesity [39]. Thereby, the complexity of the involvement of different immune cells and how they are regulated in obesity is an imperative question that remains to be answered in the field of obesity and its metabolic complications.

### How miRs are regulated

miRs regulation involves a series of steps including transcription, processing by Drosha and Dicer, transport from the nucleus to the cytoplasm by exportin, packing onto AGO proteins, stability, and turnover [40]. miRs are transcribed by RNA polymerase II [41], and this process is regulated by transcription factors like p53, MYC, ZEB, and growth factors like transforming growth factor-beta (TGF-β), platelet-derived growth factor, etc. [42–45]. Furthermore, the miRs transcription is epigenetically controlled by DNA methylation and histone acetylation [46]. The first transcribed miRs are cleaved into pre-miRs (60 nucleotides) by action Drosha (ribonuclease III enzyme) and RNA binding protein DiGeorge syndrome critical region 8 (DGCR8) [47]. After Drosha processing, pre-miRs are transferred from the nucleus to cytoplasm protein by exportin 5 (EXP5) [48]. EXP5 is universally expressed, however it is elevated in a phosphoinositide 3-kinase (PI3K) pathway in cell cycle entry [49]. After entering the cytoplasm, pre-miRs are processed by RNase III endonuclease Dicer, resulting in a mature miR (~ 22 nucleotides) duplex [50]. This miRs duplex is packed into an RNA-induced silencing complex (RISC) including the AGO [51]. Intriguingly, one strand of miRs duplex resides in AGO as miRs (the guide strand), while the other strand (the passenger strand) is degraded [52]. AGO is regulated by several pathways like mitogen-activated protein kinase-activated protein kinase 2 and AKT3 [53, 54]. The inhibition of Dicer and Drosha enzymes in human mesenchymal stem cells can reduce adipocyte differentiation, and inhibiting Drosha in 3T3-cell line also inhibits adipogenesis [55]. AT circulating miRs expression is decreased in AT-specific Dicer knockout mice, which are not effective in the processing of miRs, particularly in the AT [56].

## Do miRs regulate obesity?

It has been well established by now that miRs are extensive regulators of adipocyte differentiation, development, and function [57, 58]. However, there is a debate that miRs have both negative and positive effects on adipogenesis, which might alter the obesity state and disease outcomes. In a recent study, it has been shown that 26 miRs expressed during adipogenesis displayed expression profiles that were inversely correlated with those of adipocytes taken from ob/ob mice [59]. Since miRs have been associated with gene regulation and several other factors, they are also key to the development of obesity and other associated diseases through miR-mediated pathways. When we look at other aspects that mediate miRs, non-coding RNAs received the attention that regulates several gene expressions, which are related to different biological processes in differentiation, development, and metabolism [60]. Another study has shown that AT releases exosomal miRs that can further regulate several signaling molecules and mediate obesity [61]. Furthermore, miRs are also considered a key player in the proliferation of adipocytes, help in expansion, and have also shown an association with MS [62]. Thus, an obese environment can modulate the gene expression in several organs related to MDs, inflammation, and CVD. Many transcriptional factors, extracellular hormones, and EVs tightly control obesity and MS, but the modest information available on how miRs regulate this mechanism is the main barrier to developing therapies for obesity. However, information on miRs is appearing regularly and, in a recent study, miRs have been recognized as epigenetic regulators as single miRs can be able to regulate a variety of target genes and signaling pathways [63]. Further, another study showed that miR-14 can regulate fat cells, which exerts a suppressive effect on fat metabolism [64]. Along this line, miR-27a and miR-130a suppress adipocyte differentiation [20]. Wang et al., also demonstrated that miR-17 ~ 92 clusters were upregulated during adipogenesis [65]. Further, miR-143 has been identified as a positive regulator of human adipocyte differentiation and demonstrates a clear role of miRs in inflammation **(**Table 1**)** [66, 67]. The expression of several other miRs such as miR-146b in adipocytes was markedly increased by TNF-α and interleukin (IL)-6 [68]. The functional effect of several miRs in inflammation has been demonstrated in vivo [69, 70]. Based on this information, an altered set of differentially expressed miRs could be developed as i) biomarkers for obesity-related inflammatory disease; ii) MDs; iii) autoimmune diseases like T1 & T2DM, and iv) CVD. Now, it has been established that miRs control a variety of metabolic and signaling pathways and can be used as a targeted pharmacological approach for obesity and MDs.

**Table 1:** Role of miRs in obesity

miRs	Role in Obesity
miR-26a, miR-92a, miR-145, miR-193b, miR-450a, miR-146b-5p	Adipokine regulation
miR-16-5p, miR-214, miR-182, miR-326, miR-21, miR-143, miR-27a, miR-130a	Adipocyte differentiation
miR-1224, miR-223, miR-148a	Regulate macrophages
miR-99a	Negatively correlated with obesity
miR-222, miR-34a	Promotes insulin resistance
miR-155	Activates proinflammatory pathways
miR-326	Enhance Th17 cells in obesity

## Do adipose tissue-derived miRs alter obesity?

In this decade, miRs have emerged as key modulators of metabolic processes and play a key role in maintaining or altering energy balance, and metabolic immune homeostasis. An altered set of miRs expression has been associated with obesity, affects the function of different tissues and organs, and most importantly has been associated with low-grade chronic inflammation, which is the root cause of many diseases. Chronic inflammation in the AT is regarded as a major hallmark of obesity as adipocytes tend to show hypertrophy to store excess energy. During the hypertrophic condition, adipocytes release inflammatory molecules referred to as adipokines and cytokines. These adipokines and cytokines promote the infiltration of macrophages, T cells, and other immune cells to induce an inflammatory response in the AT. During this response period, AT macrophages as well as T cells secrete various miRs [62]. At this stage, these cells and miRs crosstalk with each other and develop an important link to impact the inflammatory state of AT and other MDs. Several of these miRs derived either from adipocytes, T cells, or macrophages have been investigated to show a significant functional role in adipocytes. For example, miR-26a, miR-92a, miR-145, and miR-193b are reported to regulate several adipokine productions including adiponectin, TNF-α, and MCP-1 in white AT (WAT) inflammation **(**Table 1**)** [66, 71, 72]. In addition, a recent study reported that AT-derived miR-1224 inhibits anti-inflammatory macrophage polarization, leading to obesity in a murine model [73]. miR-99a was reported to be negatively correlated with obesity where proinflammatory phenotypes represented decreased expression, whereas this expression was significantly upregulated for anti-inflammatory phenotype [74]. Another exosomal miR released from AT, miR-450a, was reported for increasing adipogenesis [75]. miR-222, which has been documented to be released from gonadal WAT, promotes IR resulting from obesity [76]. miRs can have a critical impact on AT by not only affecting the function of WAT and brown AT (BAT) but also having a crucial influence on the phenotypes of both WAT and BAT [77]. Taken together, it is postulated that miRs from AT act as a crucial regulator of crosstalk between other miRs derived from immune cells to endocrine hormones, which affect obesity and other MDs.

## Macrophage-associated miRs in AT during obesity

Macrophages are an important part of the innate immune system and are crucial for mediating the host defense response against inflammatory insult [78], and inflamed AT is most dominantly infiltrated by macrophages [18]. It has been established that lipopolysaccharide (LPS) and interferon-γ (IFN-γ) activate the M1 phenotype, and IL-4 and IL-13 are associated with tissue repair by activating M2 macrophages. Lean AT is dominated by M2 macrophages, whereas M2 macrophages undergo a phenotypic switch to the M1 phenotype during the progression of obesity [10]. During obesity, several macrophage-secreted factors regulate the gene expression of inflammatory cytokines and maintain a balance between M1 and M2 macrophages [79]. Hence, macrophage-regulated alteration of gene expression profile plays a crucial role in obesity and MDs. Based on recent studies that miRs are responsible for controlling gene expression, targeting macrophage-associated miRs is crucial for the attenuation of both obesity and AT inflammation.

**Fig. 1:**
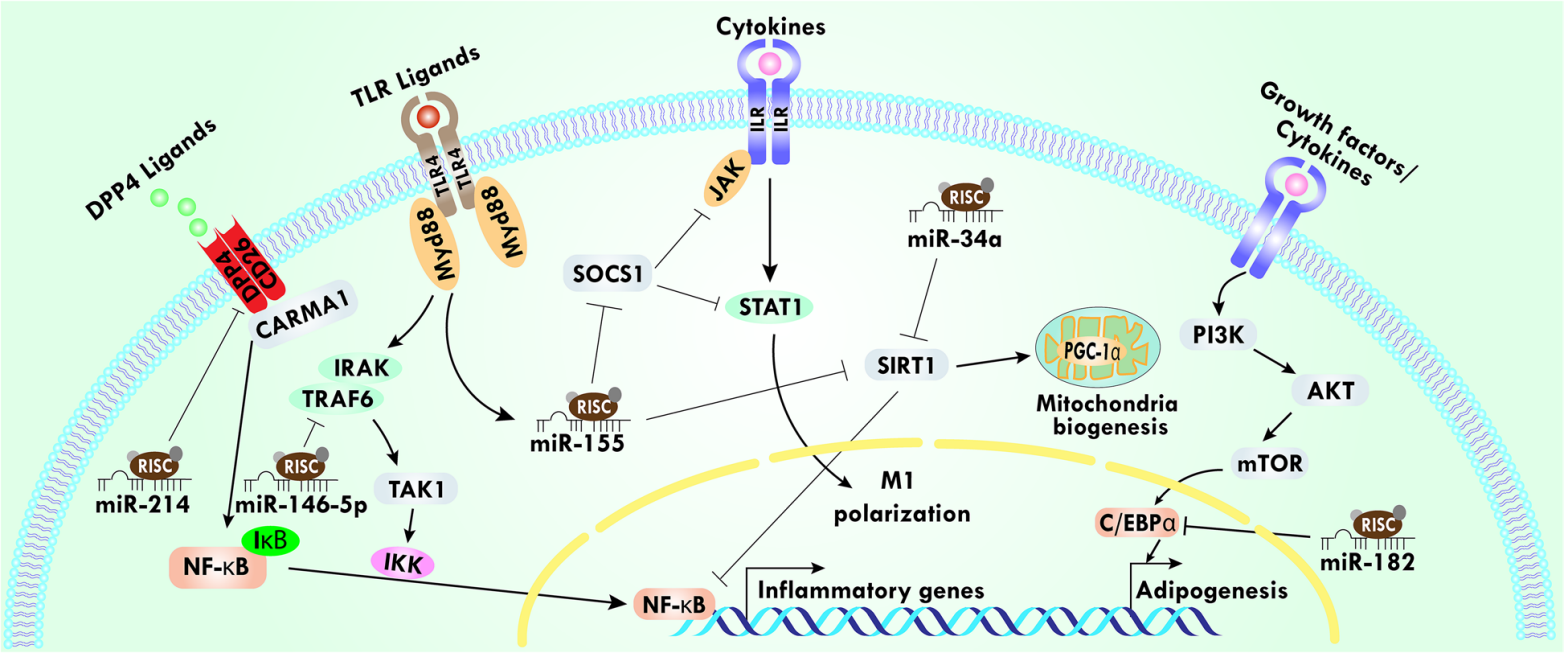
miRs mediate differential gene expression related to obesity and associated MS. miRs act on NF-κB, TLR, IL, and PI3K/AKT signaling pathways. miR-214 inhibits DPP4 that further activates the translocation of NF-κB. miR-146 inhibits TRAF6 and blocks the downstream signaling. miR-155 inhibits SOCS-1, which blocks the activity of the JAK/STAT pathway. Additionally, miR-155 along with miR-34a inhibits SIRT1 signaling and that is associated with numerous biological activities, including inhibition of NF-κB transcription factor, increasing mitochondria biogenesis by positively regulating PGC-1α. miR-182 inhibits transcriptional factor C/EBPα, which further inhibits adipogenesis. All these suggest the role of miRs in differential gene expression during obesity

**Fig. 2:**
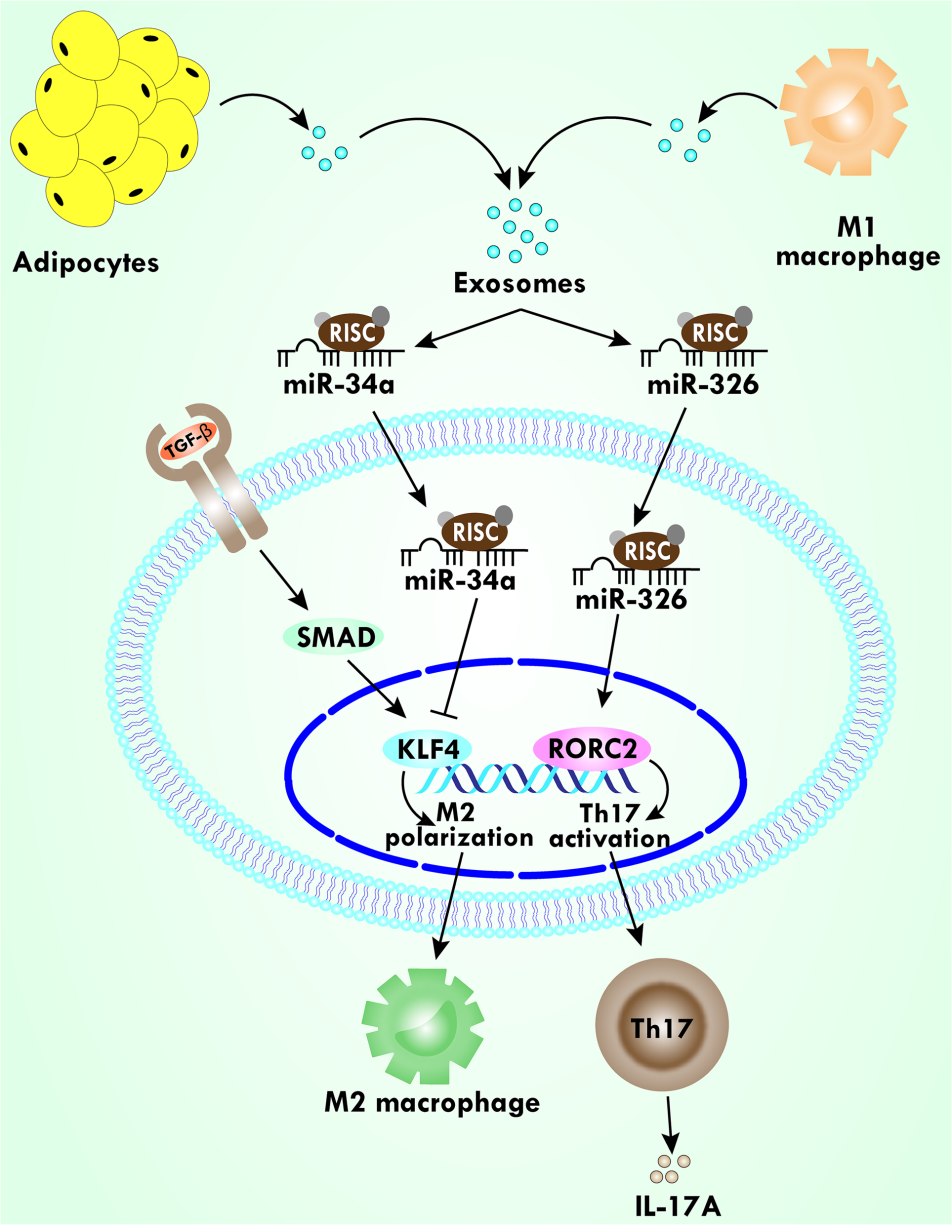
Exosomal miRs are associated with the progression of inflammation. Adipocytes-derived exosomal miR-34a inhibits the transcriptional factor KLF4 and repressing the activity of KLF4 suppresses M2 macrophage polarization. M1-macrophage-derived miR-326 increases the regulation of RORC2, leading to the regulation of the production of Th17 cells, which secretes IL-17A, and further increases the inflammation.

Recently, it has been reported that miRs are the critical regulator of innate immunity [80]. As a result, miR-155 has received a lot of attention because it is encoded by a noncoding gene known as B-cell integration cluster and released by inflammatory macrophages during various signaling, including stimulation by toll-like receptors (TLRs) ligands LPS or poly (I:C) **(**Fig. 1**)** [10, 81]. miR-155 targets the suppressor of cytokine signaling (SOCS)-1 in activated macrophages, which leads to the blockade of the negative feedback loop provided by the SOCS1 protein during the progression of inflammation **(**Fig. 1**)** [82]. In addition, SOCS1 inhibits cytokine signaling either by targeting janus kinase (JAK)-signal transducer and activator of transcription (STAT) pathway or by directly blocking TLR4 signaling pathways **(**Fig. 1**)** [83, 84]. A study by Gaudet et al., reported that deletion of miR-155 improves obesity resistance not only by reducing WAT inflammation but also biases preadipocytes to a brown-like phenotype [85]. miR-155 is shown to be responsible for the exacerbation of obesity and it also activates proinflammatory pathways [86]. Further, miR-155 expression is dependent on TNF-α, which is a proinflammatory cytokine [87]. One of the possible explanations for TNF-α dependent activation of miR-155 is that it can target sirtuin 1 (SIRT1) and suppress the expression of SIRT1 **(**Fig. 1**)** [88]. SIRT1 delineates anti-inflammatory effects and high-fat diet (HFD)-fed mice showed downregulation of SIRT1 in AT [89, 90]. Moreover, a study showed that obesity was limited by inhibiting the regulatory effects of miR-155 [85]. Taken together, these studies suggest that macrophage-derived miR-155 inhibition might be developed as a potential therapeutic target for obesity and related inflammation. We will now discuss in brief other macrophage-derived miRs that are reported to increase during obesity-related inflammation. Exosomal miRs of M1 macrophage miR-326 are associated with the elevated level of AT resident Th17 cells in obesity **(**Fig. 2**)** [91]. Likewise, another miRs derived from the exosome of the M1 macrophage, miR-16, promotes adipocyte differentiation of 3 T3-L1 **(**Table 1**)** [92]. The expression of miR-16 was elevated during adipocytes differentiation and the upregulation might be exhibited by targeting ethanolamine phosphotransferase 1 (EPT1). EPT1, a selenoprotein, is critical for phospholipid biosynthesis, but its role in obesity is not clear. Thus, the exact mechanism and the role of miR-16 during obesity require further investigation. miR-34a is upregulated in diet-induced obese mice as well as in human AT during obesity and has been correlated with IR [93]. A similar study also reported that the adipose-secreted exosomes transport miR-34a into macrophages, where miR-34a represses the M2 polarization, thus promoting obesity-induced AT inflammation **(**Fig. 2**)**. This suppression of M2 macrophage polarization was accomplished mainly by inhibiting the activity of transcription factor Krüppel-like factor 4 (KLF4) **(**Fig. 2**)**. KLF4 is crucial for adipose M2 macrophage polarization, and over-expression of KLF4 is reported to reduce the expression of proinflammatory markers associated with M1 macrophage [94]. miR-34a also targets SIRT1, which is pharmacologically beneficial for obesity disorders since it has been shown that SIRT1 activity increases oxidative metabolism as well as mitochondrial function **(**Fig. 1**)** [93, 95]. Hence, targeting miR-34a would be therapeutically promising for obesity, MDs, and related autoimmune diseases.

Another potentially important miR-223 shows a suppressive effect on the proinflammatory activation of macrophages **(**Table 1**)**, and Zhuang and co-workers reported that the knockout of miR-223 is related to increase macrophage infiltration into AT [96]. In this study, they also showed the elevated level of MCP-1 as well as proinflammatory cytokine expression in miR-223-deficient HFD-fed mice as compared to the control. Apart from this, a recent study also demonstrated that miR-223 affects the activation of macrophages by producing inducible nitric oxide synthase (NOS2) [97]. Further, miR-223 plays a multifactorial role during inflammation and involves the regulation of TLR4 and STAT signaling pathways [98]. miR-146b, which is essential for the anti-inflammatory action of adiponectin, decreases the expression of TNF-α and IL-1β by suppressing TNF receptor-associated factor (TRAF)-6 signaling, which is one of the key mediators for producing several proinflammatory cytokines regulated by nuclear factor kappa B (NF-κB) pathway **(**Fig. 1**)** [99]. In an interesting study, exosomes from M2 macrophages carry miR-148a which is shown to modulate adipocyte differentiation and regulate the macrophage-mediated immune response during infection related to obesity [100]. The immune response of miR-148a has been demonstrated by targeting phosphatase and tensin homolog deleted on chromosome 10 which leads to the alteration of several cytokines expressions [101]. This outcome was corroborated by He et al. in a pre-adipocyte miR-148a study in a rabbit model [102]. Thus, based on all these studies on macrophage-associated miRs responsible for controlling genes and the inflammatory response, it is safe to assume that targeting macrophage-associated miRs will be crucial for the attenuation of inflammatory regulation during obesity. Focusing on an altered set of macrophage-derived miRs as therapeutics might move the obesity field forward.

## Role of T cell-associated miRs in obesity and related complications

T lymphocytes (CD4 and CD8) are one of the key elements of the immune system that orchestrate highly specific and long-lasting immune responses [103]. Recent studies have demonstrated that T cells have specific functions in obese AT and miRs are engaged in the activation and function of T cells [104–106]. In the last decades, it has been accepted that T cells associated with AT miRs have potential roles in obesity [107]. Interestingly, T cells recruit macrophages to AT in obese mice, suggesting a role during obesity [13]. In this review, we will discuss only a few important T cell-associated miRs roles in obesity. Towards this, T cell-derived miR-214 shows the highest induction in AT [108]. miR-214 expression was upregulated after adipogenic differentiation in embryonic stem cells of mice, which showed that miR-214 plays a potential role in adipocyte generation [109]. Xi et al. reported that miR-214 targets the β-catenin gene (Ctnnb1), which is considered the hub gene of Wnt signaling, and primarily encodes the β-catenin protein [110]. The expression of β-catenin was increased in the AT of obese subjects as well as HFD-fed obese mice, and β-catenin is more prominent in mature adipocytes than preadipocytes. Chen and his cohort noted that deletion of β-catenin in mature adipocytes reduced macrophage accumulation in AT [111]. All these studies suggest the important link between obesity and T cell miRs in AT through β-catenin mediated pathways. Furthermore, miR-214 directly targets Ctnnb1 to inhibit the Wnt1/β-catenin signaling pathway, indicating the inhibitory role of miR-214 in obesity [110]. Obesity is characterized by a higher expression of dipeptidyl-peptidase 4 (DPP4) compared to the lean subject, thus showing more DPP4 activity in cases of obesity. The elevated plasma level of DPP4 strongly correlates with obesity-related MS [112]. DPP4, which is highly abundant in T cells, is a target gene of miR-214, suggesting that miR-214 might represent a beneficial effect by suppressing the upregulation of DPP4 **(**Fig. 1**)** [113, 114]. Taken together, miR-214 could be used as i) a potentially active biomarker for obesity and ii) targeting DPP4 to improve obesity-induced MS. However, the exact mechanism of miR-214-related inhibition in clinical samples is still unclear and needs more studies before any further conclusions can be made.

Another T cell-associated miR-182 has been shown to negatively regulate adipogenesis [115]. miR-182 targets the CCAAT/enhancer-binding protein α (C/EBPα), and this has widely been considered to be a master regulator of several genes related to adipogenesis **(**Fig. 1**)**. It has been shown that C/EBPα increased the expression of various genes characterizing the phenotypes of mature adipocytes using terminal differentiation through peroxisome proliferator-activated receptor gamma (PPAR-γ) pathways [116, 117]. Moreover, the transcriptional factor C/EBPα activation is mediated by PI3K/Akt signaling to promote the maturation of adipocytes [118]. Further, miR-182 limits the activity of C/EBPα, which is supported by a study showing that the expression of miR-182 was significantly reduced in obese humans and mice, thus demonstrating the association of miR-182 with adiposity [115]. Like miR-182, miR-326 targets C/EBPα, as the binding of miR-326 markedly declines the protein expression of C/EBPα, leading to reduced adipogenic differentiation [119]. Moreover, Feng and his colleagues reported that miR-326 plays a vital role in adipogenic differentiation, and the higher expression of miR-326 negatively regulates adipogenic differentiation [119]. A recent study reported that miR-326 is associated with the production of IL-17A by involving an increase in Th17 cells noted in AT [120]. It is possible to speculate that the deleterious effect of miR-326 might be due to the presence of miR-21, which also participates in the imbalance of Th17/Treg cells in several inflammatory diseases [91]. There is a report that miR-326 increased significantly in several inflammatories as well as autoimmune diseases, where the frequency of IL-17A-producing T cells was increased and the frequency of Treg cells was reduced. This increased level of miR-326 was associated with increased expression of nuclear hormone receptor retinoic acid receptor-related orphan receptor C2 (RORC2). In brief, RORC2 is the master regulator of transcriptional regulation of Th17 cells, leading to the generation and differentiation of IL-17A [121]. Hence, miR-326 participates in promoting the proinflammatory profile in AT during obesity through the generation of Th17 cells. Interestingly, the knockdown of miR-21 affects adiposity, weight gain, and lipid profile in a murine model, which substantially supports that miR-21 might be targeted to reduce obesity-related MS [122]. Further, inhibition of miR-21 has been shown to reduce the level of MCP-1, which is responsible for the infiltration of proinflammatory macrophages into the AT [122]. In contrast, miR-21 mimics delayed weight gain in a diet-induced obesity mouse model, which is contradictory to the other findings described above [123]. Taken together, it is suggested that T cells associated with miRs modulate obesity by altering macrophage phenotypes and other hormonal factors that control metabolism. However, further detailed studies are needed to conclude the effect of these T cell-associated miRs in the context of obesity and MDs.

## What is the potential of circulating miRs in obesity?

We have already discussed that miRs regulate gene expression through complementary binding with target mRNA at the post-transcriptional level. Mature miRs also exert their biological activity in the circulatory system as well as in different body fluids including lymphatic fluids, urine, and saliva. After processing into a mature form, miRs are either complexed with AGO proteins to stabilize or are packed within EVs [124, 125]. Recent studies suggest that these EVs contain plenty of miRs and are responsible for the transfer of miRs between cells, leading to intracellular communication along with crosstalk with several organs of the body [126]. The occurrence of miRs in the circulatory system is not clear, and evidence indicates that it might be due to disruption of the cellular membrane or another transport system in the body. In the past, various circulating miRs have been reported in the context of MDs including obesity and T2DM, and the expression of circulatory miRs is different for people with various manifestations of MS [127–129]. AT-derived circulatory miRs are considered a novel form of adipokines that possess various autocrine, paracrine, and endocrine functions [56].

AT-derived circulatory miRs are mainly released from the adipocytes and macrophages which are important for regulating several functions including energy metabolism, glucose homeostasis, and insulin sensitivity [56]. It has been shown that adipogenic miR-27a promotes M1 macrophage polarization as well as the generation of proinflammatory cytokines [130]. This stimulation, macrophage polarization, and infiltration during HFD-induced obesity by miR-27a were mediated mainly by inhibiting PPAR-γ. Another study indicated that circulating miR-27a levels were increased in obese adults and children in patients with T2DM as well as in HFD-fed mouse models [131]. Moreover, circulating miR-378 over-expression is also associated with both increased fatty acid synthesis and the size of the lipid droplet [132]. It has been shown that several proinflammatory factors such as TNF-α, IL-6, and leptin are responsible for the expression of miR-378 [133]. Furthermore, AT-derived circulating miR-92a, which is associated with BAT functions and activity, has been shown to decrease the amount of insulin expression [56]. Likewise, AT-derived circulating miR-130b level was elevated with the degree of obesity as well as MS [134]. A similar study also reported that the circulating level of elevated miR-130b was stimulated by TGF-β, and a higher level of TGF-β has been associated with increased secretion of miR-130b during obesity. Additionally, circulating miR-130b possibly affects muscle metabolism by targeting PPAR-γ coactivator-1α [134]. Another circulating miR-15a reduced obesity and T2DM, whereas an increased concentration of miR-15a has been shown in murine β-cells after administration of high glucose [135]. It has been documented that miR-15a is also associated with macrophage polarization, and its deficiency facilitates the proinflammatory activation of macrophages. However, in a different study, Lou et al. demonstrated that inhibition of miR-15a prevented the activation of NF-κB signaling by inversely regulating TNFAIP3 Interacting Protein 2 (TNIP2) expression in both in vitro and in vivo inflammatory models, although analysis for the inhibition of miR-15a in obesity-related low-grade chronic inflammation has not been reported to date [136]. Another circulating miR-132 was shown to decrease AT during obesity in these subjects as compared to lean subjects [137]. Therefore, several circulating miRs have a differential role and there is a need for an in-depth study on how these circulating miRs convey their message to macrophages, other immune cells, and adipocytes to mediate obesity and MDs in the future.

## What is the potential of miRs as biomarkers for obesity?

The clear indication of obesity-associated biomarkers may provide crucial insights into obesity-related low-grade inflammation and various metabolic pathways prevention and therapeutics [138]. These biomarkers can be used to determine the pathogenesis and phenotypes of obesity and MS. To be precise, the currently available biomarkers in the progression of obesity and related MDs are limited to date. Thus, to counter this challenge in the management of obesity, MDs include a search for innovative biomarkers, which might predict obesity’s relationship with genetics, MDs, and related low-grade chronic inflammation. The potential indicators that might be used as biomarkers should correlate with biological and metabolic parameters as well as obesity, which also represents physiological changes in tissue level and is eventually stable and specific [139]. Lately, changes in circulating or tissue-resident miRs expression with disease progression have been regarded as potential biomarkers of disease. Thus, there is growing attention in this area by several investigators, and reports are identifying many circulating miRs in distant tissues [140]. We believe that circulatory miRs possess significant potential to develop as biomarkers, as miRs have shown tremendous stability at room temperature as well as in repeated freeze-thaw cycles [141]. The stability of miRs is possibly due to association with several factors including AGO2 complexes, packaging in EVs, and lipoproteins [142, 143].

These modulations of obesity-associated circulating and tissue miRs could be used for the management of obesity. When we look at their mechanism, miRs work in several ways, and one important mode is as endocrine factors, facilitating the crosstalk between several metabolic organs and tissues [26, 138]. Next, miRs are useful for obesity management, as several miRs are differentially expressed during obesity progression. This might be useful for obesity prognosis and manipulating these miRs could result in either a worse or better obesity state. For example, two circulatory miRs, miR-17, and miR-132 were decreased in patients with obesity as compared to lean individuals [137]. Further, plasma levels of these two circulatory miRs were also reported to correlate with the regulation of several visceral AT resident miRs, thus suggesting a strong functional correlation between circulatory and tissue-resident miRs. Ortega et al. reported a significant increase in plasma miRs, including miR-140, miR-222, and miR-142 in obese persons with the morbid condition [135]. In contrast, the same study showed a decreased expression of miR-15a, miR-221, and miR-130b compared with obese to non-obese individuals. In another study, Meerson et al. showed that miR-221 positively correlated with obesity, BMI, and plasma concentrations of both insulin and glucose levels [144]. Conversely, another study reported that differentiated adipocytes obtained from obese patients reduced miR-221 expression compared to lean individuals [145]. As we know, miRs have several targets and induce many pathways, but the mechanism of miR-221 is somewhat complicated and warrants further investigation before suggesting miR-221 as a biomarker for obesity-related chronic low-grade inflammation. Moreover, Assmann et al. observed a dysregulation in the expression of miR-130a, miR-142, miR-144, miR-15a, miR-22, miR-221, and miR-29c in individuals that responded to a low-fat diet [146]. Furthermore, another cross-sectional study reported that dysregulation of several circulatory plasma miRs concentrations of miR-486, miRs-423, and miRs-130b was increased, whereas miR-28 and miR-221 were decreased during childhood obesity [147]. Other works have also demonstrated that circulatory levels of plasma miR-423, miRs-15, miRs-146, and miRs-520 were significantly decreased in individuals with severe obesity [148]. Apart from this, several circulatory miRs profiles have been reported in both pre-gestational and gestational obesity [149].

All these recent discoveries of miRs as biomarkers for obesity will pave the way for identifying individuals that might be susceptible to metabolic disorders which will, in turn, lead to complicated diseases in the future. Towards this end, it has been shown that the alteration of various circulatory miR-197, miR-320a, and miR-509 have been related to MS [150]. When we think about complicated diseases related to obesity, T2DM comes out on top, and it would be ideal to find clinically active miRs biomarkers, which might also be important for diagnosing T2DM. Interestingly, Pescador and the cohort reported that serum miR-138, miR-376a, and miR-15b act as potential biomarkers for obese diabetic patients [151]. In this study, the authors observed that both miR-138 and miR-503 can differentiate between obese-related diabetic persons and non-obese diabetic conditions/person. In addition, AT expression of miR-143 and miR-652, have been associated with lipogenesis stimulated by insulin, which suggests a role for both miRs in the development of lipogenesis and IR [152]. Other studies have also suggested the role of dysregulated miR-143 in the context of MD and obesity, and it possibly controls hepatic IR in a murine model [153]. Further, miR-26a regulates both glucose metabolism and insulin signaling, as an expression of miR-26a in the liver was decreased in obesity, which might suggest its potential role as a biomarker for obesity-related metabolic complications [154]. Another potential biomarker candidate miR-126 has shown a reduced level in serum and has emerged as a potential indicator of T2DM, which was negatively correlated with increased glucose tolerance [155]. Taken together, circulating and tissue-resident miRs field is growing in the context of obesity, metabolism, and related disease complications. Thus, these can be developed as potential biomarkers for obesity-related complicated diseases like T2DM and MS.

## What is the therapeutic potential of miRs in obesity and MDs?

In recent years, the number of human diseases has grown enormously around the world, and there is an obvious need for potential diagnostic tools and therapeutics for obesity and MDs. Due to recent discoveries discussed above, miRs can be used as potential biomarkers and can serve as excellent diagnostic tools for managing diseases including, CVD, neurological disorders, cancer, T2DM, obesity, and several other MDs. In pre-clinical and clinical trials, two miR-based therapeutic approaches have shown potential effects including miRs mimics (agonists) and anti-miR oligonucleotides (antagomirs). In addition, therapies that are based on RNA interference (RNAi) utilize small interfering RNA (siRNAs) which are similar to miRs. Both are used for the silencing of specific miRs targeted to genes. The first miR-based drug that was used in a human clinical trial was Miravirsen (antimir-122), which was used for hepatitis C virus infection and reached phase II clinical trials [156]. Interestingly, another RNAi-based drug, Patisiran, was approved by the FDA and is currently being used for hereditary transthyretin amyloidosis [157].

As mentioned earlier, miRs have a potential role in multiple pathways with pathophysiological relevance to obesity and related metabolic disorders. miR-based therapeutic approaches are currently being used in treating inflammatory diseases. This functional characterization of specific miRs and their roles in AT will help to develop AT-specific diagnostic tools for obesity-linked MDs. Several miRs are already in line to be potential therapeutic targets against obesity-associated low-grade chronic inflammation by using mimics if any significant miRs are downregulated. Currently, miRs-34 mimics are in phase I clinical trials for cancer therapy and assessment of other pathological conditions [158]. We mentioned above that AT-derived miRs have physiological as well as pathological importance in immune homeostasis. Deletion of adipocyte-derived miRs leads to the generation of MS in animal models, suggesting that AT-derived miRs can be used as potential targets for the therapeutic management of obesity-related MS [62]. Previously, it was reported that long-term inhibition of miR-21 with LNA-21 effectively suppresses miR-21 expression in the melanoma cell line, although no similar roles have been reported for obesity [159]. Until now, several AT-derived miRs with potential therapeutic activity have been investigated in animal models. One important study has shown that the knockout of miR-155 attenuated both HFD-induced IR and glucose intolerance [160]. Another study showed that blocking of miR-143 by anti-miRs in a murine model prevented obesity-induced IR [161]. The miRs field is growing very quickly and, collectively, miR-based therapeutic approaches warrant further research on several MDs to conclude the role of miRs in the context of obesity. Though the therapeutic potentiality of several miRs has been revealed by various researchers to date, there have not been any miRs-based clinical trials for obesity.

### Obesity and aging

Aging is an intricate physiological process characterized by continuing degradation, loss of function, and reduced restoration capacity of cells, tissues, and organ systems. Although aging is characterized by progressive destruction in human physiology, the process of aging relates to intricated complexity within various organs make the procedure poorly understood. Generally, with aging, adiposity as well as body fat percentage increase, and due to alterations in AT functioning, it is considered one of the vulnerable tissues in aging [162]. In addition, fat mass is mostly distributed in the abdominal region for both males and females and is associated with several complications, including CVD, T2DM, and cancer [163]. Further, the aging process is associated with elevated accumulation of senescent cells in AT, with a significant increase in cytokines, chemokines, and other factors, which are considered aging-related signals [164]. Aged AT also exhibits reduced size of the adipocyte, endothelial dysfunction, decreased vascularization, and tissue fibrosis [165]. Further, more metabolic alterations in AT occur with aging, with increased IR or changed lipolysis [166].

Growing evidence reported the role of miRs in the aging process and represents an interesting topic in aging research. A recent study reported that miR-188 functions in the aging-related metabolic phenotype, which supports the role of miRs in age-related obese conditions [167]. To date, how AT and resident immune cells effect aging is not completely understood. But, as miRs possess a crucial role in differential gene expression, they might contribute to longevity and metabolism. Moreover, changes in miRs in preadipocytes may have the effect of being self-renew [168]. This could pave the way for enlarged insulin-resistant adipocytes, leading to an inflammatory response that contributes to IR and many diseases associated with aging.

## Do miRs affect the aging process?

As we mentioned earlier, miRs target hundreds of transcripts to regulate several signaling and biological pathways including aging. Growing evidence suggests that miRs regulate age-associated processes and pathologies in many tissues including the brain, heart, bone, and muscle [169–172]. Two of these pathways, insulin-like signaling and mammalian target of rapamycin (mTOR), are associated with the cell cycle and aging. Hallmarks of aging include DNA damage response (DDR), progressive shortening of telomeres due to aging chromosomes, epigenetic changes in gene expression profile, mitochondrial dysfunction, cellular senescence, and inflammatory aging, all of which make aging a complicated process [173]. Taken together, these factors are juxtaposed with each other during the progression of aging. DDR is demonstrated as an underlying cause of cellular senescence by activating the DDR pathway through DNA double-strand breaks [174, 175]. Persistent DDR activation is also responsible for telomere shortening, leading to cellular senescence triggered by telomere signaling due to DDR [176]. Due to the complex aging process, the role of miRs in mediating complex cellular pathways play several roles to mediate aging. p53 is a crucial part of DDR and is encoded by the TP53 gene, which has received a lot of attention in the past. Studies have reported that the TP53 gene was targeted by miR-125b and miR-504 in various cell lines [177, 178]. Specifically, miR-155 targets the telomere repeat-binding factor 1 (trf1) gene, which is a component of the protein responsible for telomere maintenance [179, 180]. Another report has shown that miR-155 is mostly downregulated in vascular aging and restoration of miR-155 rescues the phenotype related to vascular aging [181]. Moreover, several miRs including miR-34a, miR-34b, and miR-34c have been reported to target different mRNAs involved in cell cycle arrest as well as CDNE2 and CDK4, and the miR-34 family is also reported to regulate telomere length [173].

**Fig. 3:**
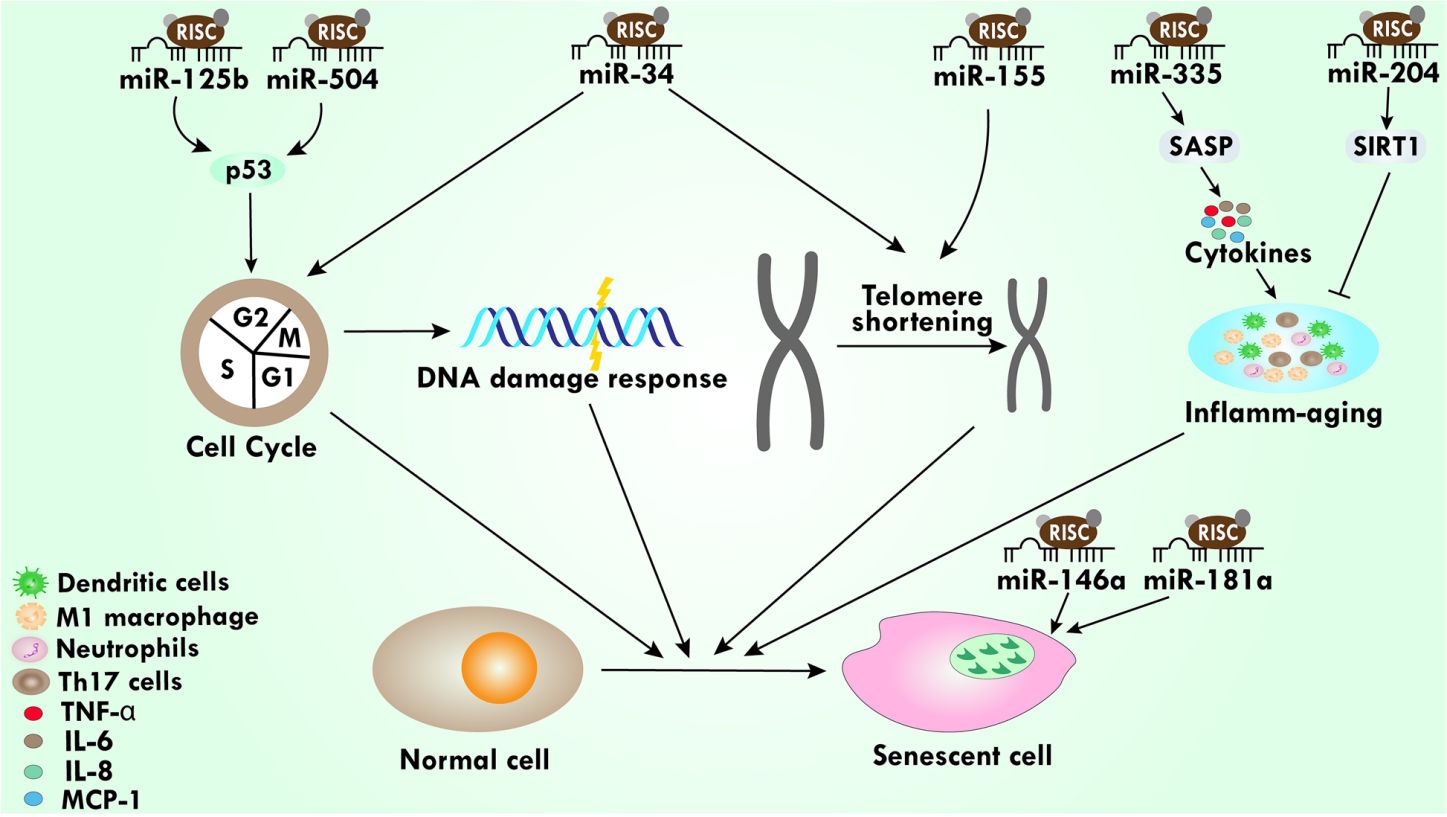
miRs play an important role in the progression of cellular senescence. miR-34 family directly affects the cell cycle, whereas miR-125b and miR-504 target p53, which is associated with cell cycle progression. Additionally, both the miR-34 family and miR-155 are associated with telomere shortening. Increased levels of miR-335 increase SASP-related factors. Several pro-inflammatory markers are associated with inflammation-aging, which is markedly decreased by SIRT1 and miR-204 upregulates SIRT-1 expression. miR-146a and miR-181a are directly associated with cellular senescence. All these events collectively pave the way for aging and suggest a crucial role of miRs in cellular senescence.

Senescence is triggered by senescence-associated secretory phenotype (SASP), which is mainly comprised of cytokines such as TNF, IL-6, IL-8, and chemoattractants like MCP-1 [182]. Cellular senescence includes several pathways such as TLR and NF-κB pathways. A study by Kabir and cohort found that the expression of miR-335 has upregulated in normal senescent cells as well as cancer cell-associated senescent fibroblast [183]. In addition, the elevated level of miR-335 has been demonstrated to increase factors related to SASP such as IL-6 and MCP-1. Interestingly, SIRT1 is a multifactorial transcriptional factor that plays a pivotal role in SASP regulation as well as cellular senescence. In an animal model, miR-204 upregulates SIRT1 expression to inhibit several cytokines including, IL-6, IL-18, and TNF-α **(**Fig. 3**)** [184].

Moreover, during inflamm-aging—a chronic, sterile, low-grade inflammation that develops with advanced age in the absence of overt infection—general levels of inflammation are elevated due to a weakened immune system. We have already mentioned that SASP has been associated with an elevated level of proinflammatory cytokines. Interestingly, it has been reported that inflamm-aging includes several pathways including TLR, NF-κB, and NOD-, LRR- and pyrin domain-containing protein 3 (NLRP3) signaling pathways [185]. In alignment with this, several inflammatory miRs are associated with modulation of the aging process, where miRs can act as an agonist for activation of the signaling cascades, leading to senescent-related low-grade systemic inflammation. It has been documented that miR-146a and miR-181a are upregulated in aging **(**Fig. 3**)** [186]. miR-146a regulates the expression of various inflammatory mediators including TRAF6 [186]. Mancini and colleagues reported that during dermal fibroblast senescence, miR-181a was upregulated, leading to the induction of cellular senescence [187]. Taken together, these studies on miRs show extensive changes in expression during the aging process and will be of growing interest to research the aging mechanism. This is a wide-open area and needs a detailed investigation for any prudent conclusions to be drawn.

## Concluding remarks

Over the last two decades, obesity and its related metabolic disorders have become a critical problem worldwide. miRs are epigenetic regulators during obesity, therefore understanding obesity and its underlying mechanisms by the expression profile of several miRs holds great promise for therapeutics. The recent discovery of miRs in biological fluids and circulating miRs profiles can be informative to differentiate between lean and obese individuals from a clinical point of view and would further enhance the efficiency of miRs-based therapeutic options. The characterization and assessment of miRs in adipose inflammation and obesity will provide a novel therapeutic target to facilitate the development of potential treatments against obesity and associated MDs. Aging is a complex process, and recent findings reported the role of miRs in aging. miRs have gained increased attention through their key roles in modulating immune functions and inflammatory responses. Taken together, the differential expression of miRs will be crucial in providing key insights into the underlying causes and mechanisms for obesity-associated aging. We believe that miRs can be developed as great therapeutics for obesity, which closely interact with MS and MDs.

## Data Availability

Not applicable.

## Abbreviations

AGO2: Argonaute-2
AT: Adipose tissue
BAT: Brown adipose tissue
C/EBPα: CCAAT/enhancer-binding protein α
CDC: Centers for Disease Control and Prevention
CVD: Cardiovascular diseases
DCs: Dendritic cells
DDR: DNA damage response
DGCR8: DiGeorge syndrome critical region 8
DPP4: Dipeptidyl-peptidase 4
EPT1: Ethanolamine phosphotransferase 1
EVs: Extracellular vesicles
EXP5: Exportin 5
HFD: High-fat diet
IFN-γ: Interferon-γ
IL: Interleukin
iNOS/NOS2: Inducible nitric oxide synthase
IR: Insulin resistance
JAK: Janus kinase
KLF4: Krüppel-like factor 4
LPS: Lipopolysaccharide
MCP-1: Monocyte chemoattractant protein-1
MHC: Major histocompatibility complex
MDs: Metabolic diseases
miRs: MicroRNAs
MS: Metabolic syndrome
NF-κB: Nuclear factor kappa B
NLRP3: NOD-, LRR- and pyrin domain-containing protein 3
PI3K: Phosphoinositide 3-kinase
PPARγ: Peroxisome proliferator-activated receptor-γ
RNAi: RNA interference
RORC2: Retinoic acid receptor-related orphan receptor C2
SASP: Senescence-associated secretory phenotype
siRNAs: Small interfering RNAs
SIRT1: Sirtuin 1
SOCS1: Suppressor of cytokine signaling-1
STAT: Signal transducer and activator of transcription
T1 & T2DM: Types 1 and 2 diabetes mellitus
TGF-β: Transforming growth factor-β
Th: T helper cells
TLRs: Toll-like receptors
TNF-α: Tumor necrosis factor-α
TNIP2: TNFAIP3 Interacting Protein 2
TRAF6: TNF receptor-associated factor-6
Tregs: Regulatory T cells
WAT: White adipose tissue
WHO: World Health Organization
